# Below-elbow or above-elbow cast for conservative treatment of extra-articular distal radius fractures with dorsal displacement: a prospective randomized trial

**DOI:** 10.1186/s13018-019-1530-1

**Published:** 2019-12-30

**Authors:** Gaetano Caruso, Francesco Tonon, Alessandro Gildone, Mattia Andreotti, Roberto Altavilla, Alessandra Valentini, Giorgia Valpiani, Leo Massari

**Affiliations:** 1 Orthopedic and Traumatology Unit, Sant’Anna University Hospital of Ferrara, Via Aldo Moro 8, 44124 Cona, Ferrara, Italy; 2 Department of Biomedical and Speciality Surgical Sciences, University of Ferrara, Via Luigi Borsari 46, 44121 Ferrara, Italy; 3 Department of Morphology, Surgery and Experimental Medicine, University of Ferrara, Via Luigi Borsari 46, 44121 Ferrara, Italy; 4 Department of Statistics, University of Bologna, Via Zamboni 33, 40126 Bologna, Italy; 5 Research Innovation Quality and Accreditation Unit, Sant’Anna University Hospital of Ferrara, Via Aldo Moro 8, 44124 Cona, Ferrara, Italy

**Keywords:** Extra-articular distal radius fractures with dorsal displacement, Above-elbow cast, Below-elbow cast, Conservative management

## Abstract

**Background:**

Distal radial fractures are common traumatic injuries, but their management remains controversial also in case of conservative treatment regarding the type of immobilisation. Hence, we conducted a two-arm, parallel-group, prospective randomised trial to compare the capacity of long casts (above-elbow) and short casts (below-elbow) to maintain the reduction of extra-articular distal radius fractures with dorsal displacement (AO/OTA classification: 2R3A2.2).

**Methods:**

Seventy-four eligible patients with AO/OTA 2R3A2.2 fractures treated with closed reduction and cast immobilisation were randomised to the long cast group (*n*°= 37) or to the short cast group (*n*°= 37). Baseline radiological parameters, radial inclination (RI), radial height (RH), ulnar variance (UV) and palmar tilt (PT) were taken, and compared with clinical (DASH, Mayo Wrist and Mayo Elbow) and radiological scores taken at 7–10 days, 4 weeks and 12 weeks. Furthermore, to evaluate correlations between radiological parameters and functional outcomes, patients were divided into two groups according to whether or not their radiological parameters at Follow-ups 2 and 3 were acceptable, i.e. within the range 11–12 mm for RH, 16°–28° for RI, − 4–+ 2 mm for UV and 0°–22° for PT.

**Results:**

Patient demographic and baseline radiological parameters were similar between groups. At follow-up, there were no statistically significant differences between the two types of cast in terms of RI, RH, UV or PT, or Mayo wrist or DASH scores. Short cast group patients displayed better Mayo elbow score at follow-up 2 (4 weeks), but this difference was no longer statistically significant at follow-up 3 (12 weeks). No statistically significant differences in clinical outcomes were found between patients who presented acceptable radiographic parameters at follow-up and those who did not.

**Conclusion:**

As there were no significant differences between short casts and long casts in terms of fracture reduction maintenance or clinical outcomes, short casts are an effective method of post-reduction immobilisation in AO/OTA 2R3A2.2 fracture of the radius. Radiological parameters outside the range conventionally considered acceptable do not preclude a satisfactory clinical outcome.

**Trial registration:**

ClinicalTrials.gov PRS, NCT04062110. Registred 20 August 2019.

## Introduction

Distal radial fractures (DRF), whose main characteristics were first described by the Irish surgeon Sir Abraham Colles in 1814 [1], are common traumatic injuries. Distal radius fractures are one of the most common types of fractures accounting up to 18% of all fractures in the adults; in 2001, it was reported that 640,000 people in the USA were treated for wrist fracture [2]. DRFs are most often encountered in two distinct groups of patients, namely young people with good bone density mineralization, who typically receive this injury via high-energy trauma; and the elderly, predominantly females with poor bone density mineralization [3]. In this latter group, wrist fractures often occur through low-energy trauma events such as falling onto the palm with the wrist extended [4, 5].

Despite its frequency, management of DRF is still extremely variable; even today, there are no clear indications as to the best treatment (conservative or surgical) for the different fracture subtypes [6]. In particular, one aspect that remains controversial is the choice of plaster cast type to be used in cases in which conservative treatment is considered appropriate [7]. Early works by Sarmiento et al. [8] and Bunger et al. [9] suggest that an above-elbow plaster cast is necessary to maintain good fracture reduction, but other authors have highlighted the fact that immobilising the elbow joint is not always necessary in this clinical context, and that a splint or so-called below-elbow cast (antebrachial–metacarpal) is sufficient to treat some forms of DRF [10–12]. To date, however, there is no clear definition of the precise indications for short casts in treatment.

Hence, the aim of this prospective randomised study was to shed more light on the issue by comparing the capacity of long plaster casts (above-elbow, LC) and short plaster casts (below-elbow, SC) to maintain the reduction of extra-articular distal radius fractures with dorsal displacement (2R3A2.2, according to the AO/OTA classification) [13]. The initial hypothesis was that the short cast would be equally as effective as the long cast in treating this type of fracture. The secondary objective of the study was to determine whether or not there is a direct correlation between radiological parameters and functional outcomes in such patients.

## Patients and methods

**Fig. 1:**
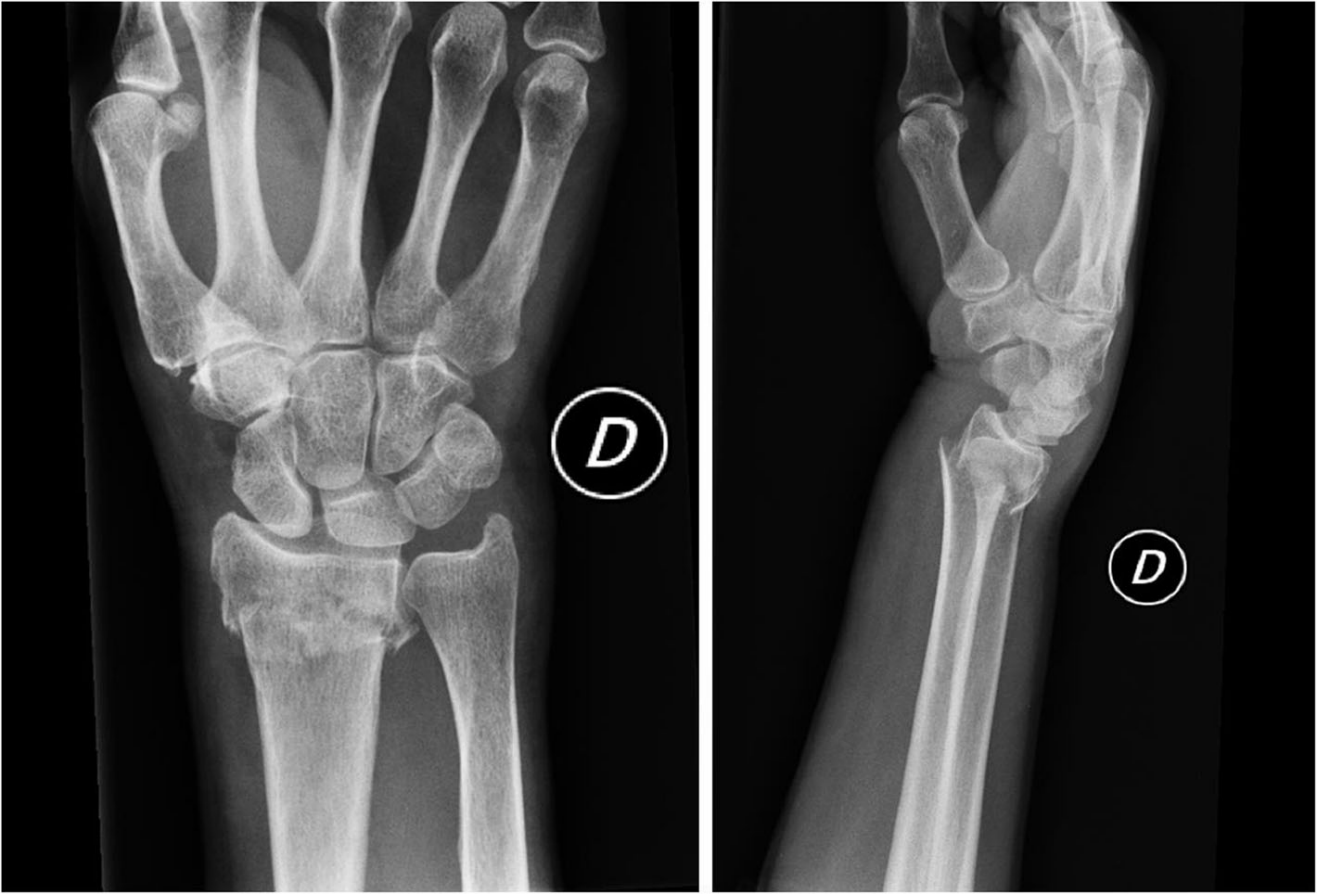
Postero-anterior and lateral view radiographs showing extra-articular fractures of the distal radial epiphysis with dorsal displacement (type 2R3A2.2 according to the AO/OTA classification)

We conducted a two-arm, parallel-group, prospective randomised trial to compare short cast (SC) and long cast (LC) treatment of distal radial metaphyseal fractures. Patients aged 18 years and over with extra-articular fractures of the distal radius and dorsal displacement (type 2R3A2.2 according to the AO classification), recruited between June 2017 and November 2018 and scheduled for conservative treatment were enrolled (Fig. 1) [13]. Patients with open fractures, extra-articular distal radius fracture with volar displacement, a history of allergy to the cast material, and those scheduled for surgical treatment because of patients’ refuse of conservative treatment were excluded, as were patients aged under 18.

Patients seen by the Orthopaedics team of our Emergency Department (ED) who met the inclusion criteria were asked to provide informed written consent, and allocated to two groups, short cast (SC) and long cast (LC).

Web-based simple randomization was performed by an investigator not involved in the trial according to a computer-generated list with an allocation ratio of 1:1 (experimental-to-active control group).

Closed reduction of the fracture was performed in all cases, and the fracture was subsequently immobilised via short cast or long cast, according to the experimental group assigned (SC or LC). For analgesia, we used the haematoma block technique with 8 cc of 1% lidocaine.

In both groups, the cast was constructed by the same sequence of bandages and the wrist immobilisation position was the same, with pronated forearm and 20° wrist flexion and ulnar deviation.

**Fig. 2:**
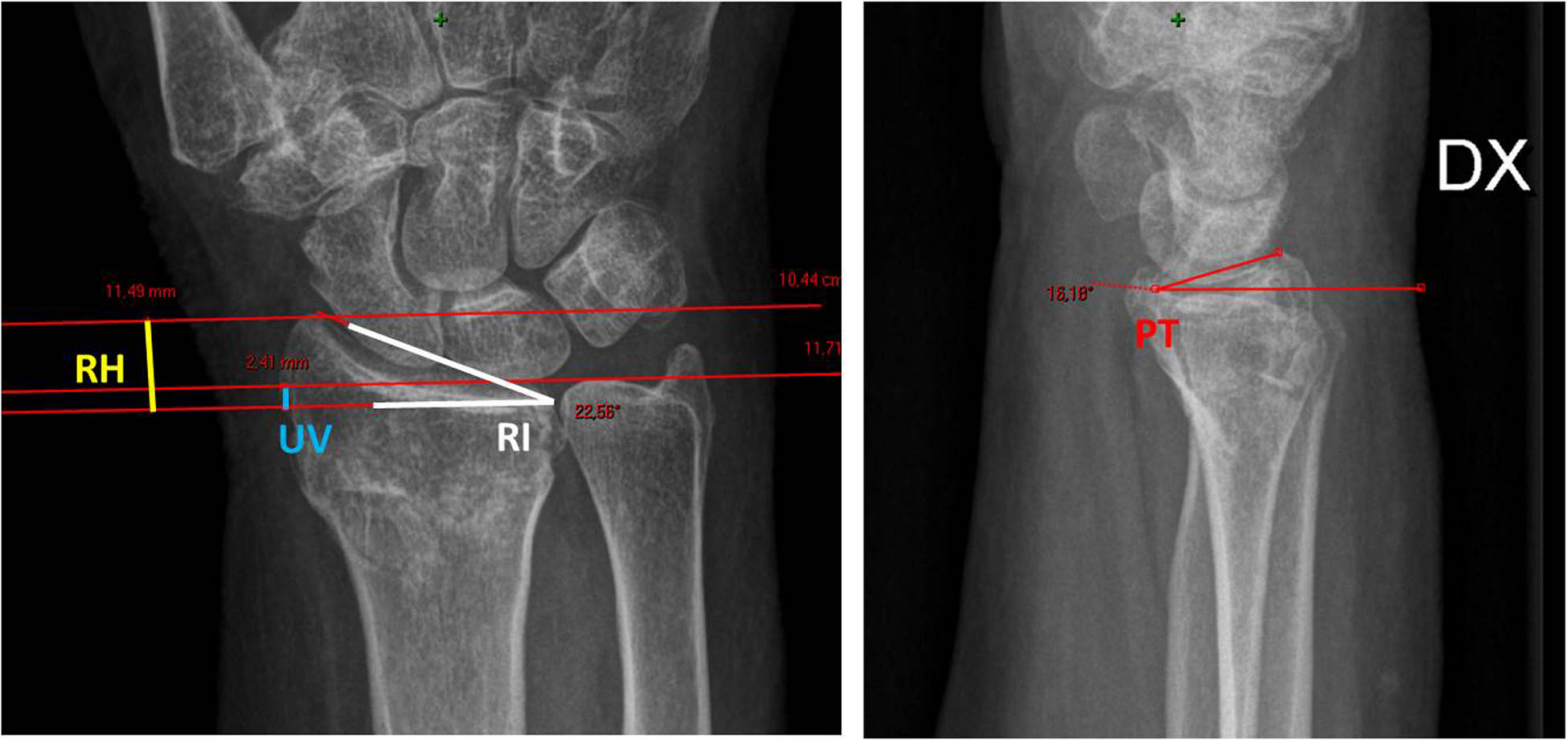
Standard (postero-anterior and lateral view) radiographs showing normal measurements of the distal radius. Palmar tilt (PT) of the radius can be measured by obtaining the angle of intersection between a line drawn tangentially across the most distal points of the radial articular surface and a perpendicular to the midshaft of the radius. Radial inclination (RI) is the angle of the distal radial surface with respect to a line perpendicular to the shaft. Radial height (RH) is the distance between two parallel lines drawn perpendicular to the long axis of the radial shaft, one from the tip of the radial styloid and the other from the ulnar corner of the lunate fossa. Ulnar variance (UV) refers to the relative lengths of the distal articular surfaces of the radius and ulna.

The reduction obtained was checked on x-rays, which were then used to calculate the following baseline radiological parameters: radial inclination (RI), radial height (RH), ulnar variance (UV) and palmar tilt (PT) (Fig. 2).

Clinical and radiological follow-up was performed at 7–10 days (follow-up 1), 4 weeks (follow-up 2) and 12 weeks (follow-up 3). X-rays at follow-up 1 were taken in the cast. Follow-up x-rays were examined to check maintenance of the reduction, and any patients presenting a degree of fracture displacement warranting surgical treatment at follow-up 1 were excluded. The clinical exam at follow-ups 2 and 3 included the Disabilities of the Arm, Shoulder and Hand questionnaire (DASH), Mayo Wrist and Mayo Elbow scores. Baseline radiological parameters were compared with those calculated for follow-ups 1, 2 and 3. In order to minimise inter-operator variability, all radiological measurements for each patient were made by the same operator using the Picture Archiving and Communication System (PACS).

In addition, to evaluate correlations between radiological parameters and functional outcomes at the end of follow-up, we allocated all patients to one of two groups according to whether or not radiological parameters at follow-ups 2 and 3 were acceptable as defined by Mann et al. [14]. Specifically, parameters considered acceptable were RH of 11–12 mm, RI of 16°–28°, UV of between − 4 and + 2 mm and PT of between 0° and 22°.

The study was approved by the Local Ethics Committee, and data collection and analysis were performed in compliance with the Declaration of Helsinki.

### Sample size

To calculate sample size, we used data from our pilot study (16th EFORT Annual Congress, 28 May 2015, Poster Session EFFORT 2015-2005).

Considering as primary outcome, we found a radial inclination effect size (*d*) of 0.69 in subjects with distal radius fractures. We calculated this value comparing the maintain of the reduction of extra-articular distal radius fractures in long cast group of 20.9 ± 4.0 (degrees) to 18.4 ± 3.2 (degrees) in short cast (control group). Given equal allocation (1:1) between short and long cast treatment arms, and using 80% power and alpha of 5%, we would need 66 subjects (33 with short cast treatment + 33 long cast treatment) to complete the study. Conservatively, we expect a 12% rate of dropout, thus the sample size will be increased by 12% to 74 subjects (37 in each group).

### Statistical analysis

Statistical analyses were performed according to the intent-to-treat paradigm, which means all patients were analysed according to the treatment group to which they were randomised. Continuous variables were presented as means and standard deviations (SD) for normally distributed data, and medians and interquartile ranges (IQRs) for non-normally distributed data, while categorical variables were presented as frequencies and percentages. The *t* test, Fisher’s exact test or Pearson chi-square test were applied, depending on the nature of the variable. To compare the difference between SC and LC groups at each time-point, *t* tests were performed for continuous variables, whereas non-parametric Wilcoxon rank-sum tests were applied to non-normally distributed variables. The Friedman test was performed for clinical parameters (i.e. RI, RH, UV and PT) over time within SC and LC groups. Wilcoxon post-hoc analysis was conducted thereafter. All statistical analyses were performed using Stata 12 (Stata Statistical Software: Release 12. College Station, Texas). All test were two-sided. The level of significance was set at a *p* value of < 0.05.

## Results

**Fig. 3:**
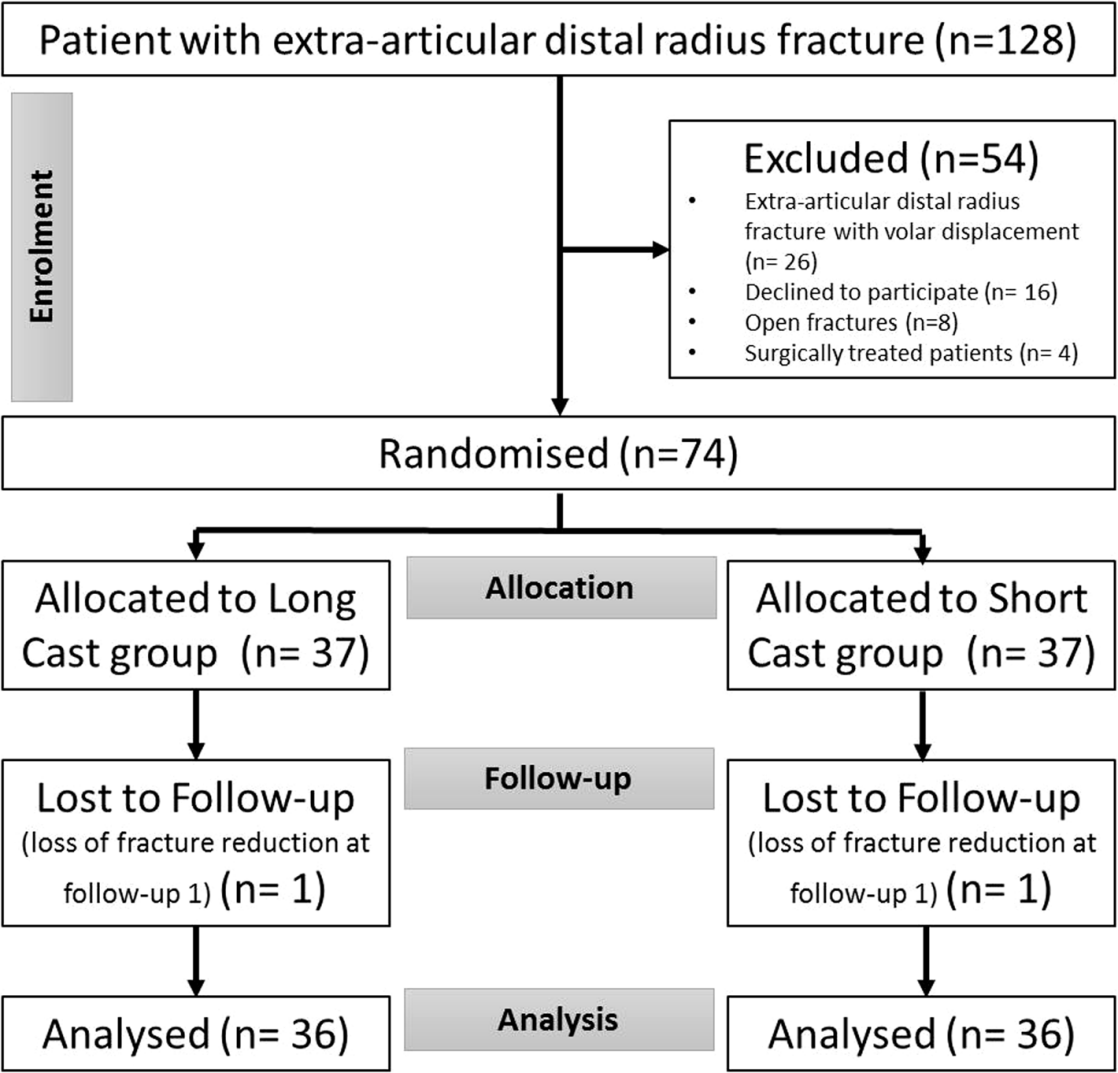
Flow chart describing the inclusion and exclusion criteria of the study

Seventy-four patients who met the inclusion criteria were randomised to the two treatment groups. Two patients (one from group SC and one from group LC) were subsequently excluded due to significant loss of fracture reduction at follow-up 1, and consequent surgical intervention. The remaining 72 patients, treated with either SC or LC, completed the radiological and clinical follow-up as described above (Fig. 3).

The demographic features of the two groups are summarised in Table 1. There were no statistically significant differences between groups in either sex (*p* = 0.999), age (*p* = 0.434) or fracture side (*p* = 0.804) (Table 1). Furthermore, no statistically significant differences were found between the two groups in terms of baseline RI, RH, UV and PT, measured from x-rays taken in ED after fracture reduction and cast application (Table 2).

**Table 1:** Patient characteristics of the total population

	Total population (*N* = 72)	Short cast (*N* = 36)	Long cast (*N* = 36)	*p* value
Age at time of the injury (years) Mean ± sd	70.9 ± 14.8	72.3 ± 14.4	69.5 ± 15.2	0.434^a^
Gender Female *N* (%)	65 (90.3%)	33 (91.7%)	32 (88.9%)	0.999^b^
Side Rigth *N* (%)	25 (34.7%)	13 (36.1%)	12 (33.3%)	0.804^c^

Variables age, gender, side are not statistically associated to short/long cast variable

^a^*t* test

^b^Fisher’s exact test

^c^Pearson Chi-square test

**Table 2:** Radiological measurements at follow up

	Baseline		7–10 days		4 weeks		12 weeks	
	Short cast	Long cast	Short cast	Long cast	Short cast	Long cast	Short cast	Long cast
Radial inclination (°)	25 [22 27]	23.5 [22 26]	23.5 [20.5 25]	21 [18 24]	22 [19 25]	21 [18 23]	22 [19 24.5]	21 [17.5 23]
Radial height (mm)	10 [9 11]	10 [9 12]	9 [8.5 10]	9 [7 12]	9 [8 10]	9 [6.5 10]	9 [7 10]	8 [6 10]
Ulnar variance (mm)	0 [− 1.25 0]	0 [− 2.25 0.5]	0 [− 1 1]	0 [− 2 1]	0 [0 1.25]	0 [− 1 2]	0 [0 1]	0 [− 0.5 2.25]
Palmar tilt (°)	9.5 [3 12]	9.5 [5.5 14.5]	6.5 [0 10.5]	5 [0 10]	2.5 [− 1.5 9]	0 [− 1.5 7.5]	0 [− 4.5 8]	0 [− 4 7.5]

Data are represented by median [25%percentiles 75%percentiles]

All comparisons between Short and Long cast groups are not significantly different (two-sample Kolmogorov-Smirnov test)

In regards to fracture reduction maintenance, there were no statistically significant differences between the two types of cast in terms of RI, RH, UV or PT at either follow-up 1 or follow-ups 2 and 3 (Table 2). Neither were there any statistically significant differences between the two groups in either Mayo Wrist (*p* = 0.999; *p* = 0.825) or DASH scores (*p* = 0.999; *p* = 0.615) at follow-ups 2 and 3. In fact, the only statistically significant difference between the two groups was the Mayo Elbow score at follow up 2; specifically, SC patients had a better score than those treated via LC (*p* < 0.001). Nonetheless, this difference was no longer statistically significant at follow-up 3 (*p* = 0.999) (Table 3).

**Table 3:** Clinical outcomes at follow up

	4 weeks		*p* value	12 weeks		*p* value
	Short cast	Long cast		Short cast	Long cast	
Mayo wrist score	1 [1 1]	1 [1 1]	0.999	3 [3 4]	4 [3 4]	0.825
DASH score	71.7 [67.1 76.3]	72 [67.45 76.3]	0.999	0.8 [0 2.1]	1.7 [0 2.5]	0.615
Mayo elbow score	4 [4 4]	3 [2 3]	< 0.001	4 [4 4]	4 [4 4]	0.999

Data are represented by median [25%percentiles 75%percentiles]. Two-sample Kolmogorov-Smirnov test

Concerning the secondary aim of the trial, i.e. to discover any direct correlation between radiological parameters and functional outcomes in patients with AO 2R3A2-2 fracture, we observed that after the first 4 weeks, patients with radiographic measurements considered acceptable as defined by Mann et al. [14] were 83% for radial inclination, 72% for ulnar variance, 47% for palmar tilt and 15% for radial height. The values worsen further during the follow-up, in fact at 12 weeks patients with radiographic measurements considered acceptable were 80.5% for radial inclination, 69% for ulnar variance, 44% for palmar tilt and 8% for radial height. Nevertheless no statistically significant differences in clinical outcomes (measured by Mayo wrist score and DASH score) were found between patients who presented acceptable radiological parameters [14] at follow-ups 2 and 3 and those who did not (Table 4).

**Table 4:** Clinical outcomes for patients with radiological measurements Out and In the respective reference range at follow up

		Radial inclination (°)		Radial height (mm)	
		OUT (12 patients)	IN (60 patients)	OUT (61 patients)	IN (11 patients)
4 weeks	Mayo wrist score	1 [1 1]	1 [1 1]	1 [1 1]	1 [1 2]
	DASH score	72.2 [69.2 78.9]	71.7 [65.9 75.5]	71.7 [68.3 75.9]	67.5 [60 77.5]
		OUT (14 patients)	IN (58 patients)	OUT (66 patients)	IN (6 patients)
12 weeks	Mayo wrist score	4 [3 4]	3.5 [3 4]	4 [3 4]	3.5 [3 4]
	DASH score	1.3 [0 2.5]	0.8 [0 2.5]	0.9 [0 2.5]	0.4 [0 1.7]
		Ulnar variance (mm)		Palmar tilt (°)	
		OUT (20 patients)	IN (52 patients)	OUT (38 patients)	IN (34 patients)
4 weeks	Mayo wrist score	1 [1 1]	1 [1 1]	1 [1 1]	1 [1 1]
	DASH score	70 [60 78.9]	72 [68.2 75.9]	71.7 [68.3 75.9]	71.9 [66.7 76.7]
		OUT (22 patients)	IN (50 patients)	OUT (40 patients)	IN (40 patients)
12 weeks	Mayo wrist score	4 [3 4]	3.5 [3 4]	3 [3 4]	4 [3 4]
	DASH score	0.8 [0 2.5]	1.3 [0 2.5]	0.8 [0 2.1]	1.3 [0 2.5]

Data are represented by median [25%percentiles 75%percentiles]

Reference range of patient for continuous parameters: radial inclination = 16°–18°; radial height = 11-12 mm; ulnar variance= − 4–+ 2 mm; palmar tilt = 0°–22°

## Discussion

Even after the publication of the AAOS guidelines on conservative versus surgical treatment of DRF in 2009 [15], there is still considerable debate surrounding the issue, and it is unclear which immobilisation technique is the best in cases in which conservative treatment is opted for. However, our finding is that there is no significant difference between short casts and long casts in terms of the maintenance of fracture reduction or clinical outcomes in extra-articular DRFs. However, the fact that short casts do not immobilise the elbow means that short-term clinical outcomes related to this joint may be better in this group, as the pronation-supination and flexion-extension typical of long casts is avoided.

Long casts have been used in orthopaedics for many decades, and even as far back as 1938, Lambrinudi, published in the Guy’s Hospital Gazzette, pointed out that the Colles fracture was caused by wrist trauma with the forearm in supination, and should therefore be immobilised by means of a long cast with pronation of the forearm [16]. However, this conclusion was disputed by Sarmiento et al., who stated that this immobilisation position could provoke brachioradial muscle tension, which, upon contraction, could lead to loss of fracture reduction [17]. In the attempt to resolve the issue, the same authors proposed immobilising the Colles fracture with above-elbow cast with the elbow in flexion, the forearm in supination and the wrist in moderate ulnar and volar flexion. In support of this approach, they published an article in which they stated that they had achieved good-to-excellent results in 82% of patients treated via this method, in addition to a reduced incidence of fracture reduction loss [8].

Following a prospective study comparing the different types of DRF immobilisation, Wahlstrom argued that elbow immobilisation was necessary to avoid loss of fracture reduction. However, and in contrast with Sarmiento et al., that author noted that a plaster cast in pronation was associated with a lower incidence of loss of fracture reduction in comparison with one in supination or in the midway position [17, 18]. Bunger et al., on the other hand, published the outcomes of a prospective randomised trial comparing functional bracing in supination and dorsal plaster immobilisation of DRF; the former treatment involved an above-elbow functional brace in supination, while the second a below-elbow plaster splint. They noted a statistically significant difference in fracture reduction between the two techniques, and therefore concluded that the functional brace in supination was preferable to any other immobilisation technique due to both its capacity to maintain fracture reduction and the excellent clinical results achieved [9]. Fernandez et al. also promoted above-elbow casts in an article published in 2005, maintaining that pronation-supination movement of the forearm was to be prevented in Colles fractures [19].

Fuelling the opposite side of the debate was a prospective study by Pool, published in 1973, in which it was stated that there was no benefit to immobilising Colles fractures with an above-elbow cast; indeed, that author concluded that above-elbow immobilisation yielded worse clinical outcomes [10]. Stewart et al., who conducted a prospective randomised trial on 243 patients with Colles fracture, broadly agreed with their stance, having found no statistically significant difference in either maintenance of fracture reduction or clinical outcomes between a below-elbow plaster cast, an above-elbow cast brace in supination and a below-elbow cast brace [20]. Subsequently, Tumia et al. stated that there was no statistically significant difference between a prefabricated functional brace (the Aberdeen Colles’ fracture brace) and a conventional Colles plaster cast in DRF treatment in terms of either clinical or radiological outcomes [11]. This was supported by Bong et al., whose prospective randomised trial demonstrated that there were no statistically significant differences in fracture reduction loss between a short-arm radial gutter splint and a sugar-tong splint; in fact, they concluded their article by recommending a short-arm radial gutter splint in the treatment of compound fractures of the distal radius [21].

Moreover, Gamba et al. published a prospective randomised trial comparing short- and long-arm casts in DRF treatment in which they detected no statistically significant differences between the two groups of patients in terms of either clinical outcomes or maintenance of fracture reduction; in fact, they showed that patients treated by means of a short cast displayed a reduced loss of reduction in terms of palmar tilt [22]. Recently, Park et al. also conducted a prospective randomised trial that indicated no substantial difference between short-arm and long-arm plaster casts in the treatment of stable DRF in patients of over 55 years of age; indeed, the only difference in radiological parameters between the two groups that reached statistical significance was volar tilt. However, according to the authors, this was no longer an issue at follow-up, 24 months after the fracture, as there were no differences in clinical outcomes between the two groups [12]. Finally, in their retrospective study, Maluta et al. reviewed 297 patients affected by DRF who required manipulation and were immobilised with an above-elbow cast or a below-elbow cast. They focused on maintenance of reduction, in term of radial height, radial inclination and volar tilt and they concluded that above- and below-elbow casts had comparable performance in maintaining reduction of manipulated DRF [23].

The prospective studies described above differ from ours regarding the type of immobilisation [11, 20–22], often difficult to achieve in a common orthopaedic emergency room, and for the patients selection [12].

In particular, Tumia et al. compared a conventional Colles’ plaster cast (control group) to a prefabricated functional brace (the Aberdeen Colles’ fracture brace) [11]; Stewart made a comparison between a conventional Colles’ cast to an above-elbow cast-brace with the forearm in supination and a below-elbow cast-brace [20]; Bong et al. compared a short-arm radial gutter splint with a sugar tong splint [21]; Gamba et al. compared an above elbow cast to a below elbow cast; however in their work, all patients were treated with an above elbow cast initially, then converted to a below elbow cast in a group of patients [22]. In our study, all patients were immediately treated with a different type of cast in relation to the group to which they were allocated. Finally, Park et al. considered patients older than 55 years, while we considered patients aged 18 years and over [12].

Our finding that there were no statistically significant differences in clinical outcome between patients who had and did not have acceptable radiological parameters is in agreement with the conclusion by Jaremko et al. that the normal range of radiological parameters and the various indices of acceptability in the literature are inefficient, or even useless when dealing with DRF in elderly patients [24]. Joung et al., Beumer et al. and Anzarut et al. too demonstrated that there is no correlation either across multiple measures of radiological deformity and different clinical outcomes, or when analyses were limited to deformities defined only by “unacceptable” palmar tilt [25–27]. Egol et al. also reached the conclusion that the radiological parameters considered in cases of DRF are not always correlated with functional outcomes in the elderly, following a retrospective review of elderly patients with displaced DRF treated with or without surgical intervention published in 2010. Indeed, they noted no statistically significant differences in clinical outcome between the two groups, despite the surgical patients displaying significant better radiological parameters [28].

Our study did have some limitations. First and foremost, differences in radiological technique and human error could have represented sources of bias. Furthermore, the short duration of follow-up (12 weeks) did not enable us to assess any cases of post-traumatic arthrosis that may have arisen.

## Conclusions

This prospective randomised trial demonstrates that patients treated with short casts have comparable radiological and functional scores to those treated using long casts, with fewer complications secondary to immobilisation of the elbow joint. Hence, short casts are an efficacious method of post-reduction immobilisation in extra-articular metaphyseal fracture of the distal radius, and radiological parameters outside the range conventionally considered acceptable do not preclude a satisfactory clinical outcome in elderly patients.

## Data Availability

The datasets used and/or analysed during the current study are available from the corresponding author on reasonable request.

## Abbreviations

AO/OTA: AO-Müller/Orthopaedic Trauma Association Fracture and Dislocation Classification
DASH: Disabilities of the Arm, Shoulder and Hand questionnaire
DRF: Distal radial fractures
ED: Emergency department
IQRs: Interquartile ranges
LC: Long plaster casts (above-elbow)
PACS: Picture Archiving and Communication System
PT: Palmar tilt
RH: Radial height
RI: Radial inclination
SC: Short plaster casts (below-elbow)
SD: Standard deviation
UV: Ulnar variance
